# The Role of Gene Expression Profiling in the Management of Cutaneous Squamous Cell Cancer: A Review

**DOI:** 10.3390/cancers16233925

**Published:** 2024-11-23

**Authors:** Ryan A. Durgham, Joel Badders, Shaun A. Nguyen, Lindsay Olinde, John Pang, Cherie-Ann O. Nathan

**Affiliations:** 1 Department of Otolaryngology Head and Neck Surgery, Medical University of South Carolina, Charleston, SC 29425, USA; durgham@musc.edu (R.A.D.); nguyensh@musc.edu (S.A.N.); 2 Department of Otolaryngology Head and Neck Surgery, Louisiana State University Health Sciences Center, Shreveport, LA 71103, USA; joel.badders@lsuhs.edu (J.B.); lindsay.olinde@lsuhs.edu (L.O.); john.pang@lsuhs.edu (J.P.)

**Keywords:** cutaneous squamous cell carcinoma, gene expression profiling, skin cancer, prognostics, personalized medicine

## Abstract

Cutaneous squamous cell carcinoma (cSCC) is the second most common form of skin cancer, with an increasing global incidence. While most cases are successfully treated with surgical excision, a subset can metastasize, leading to significant morbidity and mortality. Current staging systems based on clinical and histopathological features have shown limitations in accurately predicting metastatic risk. This review examines the role of gene expression profiling (GEP), particularly the 40-gene expression profile (40-GEP) test, in improving risk stratification and management of cSCC. We assess the prognostic value of the 40-GEP test, its integration with current staging systems, and its impact on clinical decision-making. Recent studies suggest that incorporating GEP results with traditional staging methods can enhance the identification of high-risk patients, potentially leading to more personalized treatment strategies. The review also explores the challenges of implementing GEP in routine clinical practice, including cost-effectiveness considerations and the need for standardization. Finally, we discuss the implications for future cSCC management and highlight areas for further research. As molecular profiling techniques continue to evolve, GEP represents a promising approach to optimizing care for cSCC patients, aligning with the growing emphasis on personalized medicine in oncology.

## Simple Summary

Cutaneous squamous cell carcinoma (cSCC) is a common type of skin cancer. While most cases are curable, some can spread to other parts of the body, becoming life-threatening. Currently, physicians use physical features of the tumor to predict which cases might spread, but this method is not always accurate. A new test called gene expression profiling (GEP) looks at the activity of certain genes in the tumor to better predict which cases are high-risk. This review examines a specific 40-gene test for cSCC, evaluating how it works and reporting accuracy in predicting metastasis and potential impacts on patient care. We found that GEP can improve risk prediction when used alongside current methods. This could help doctors make better decisions about treatment and follow-up care, potentially improving outcomes for patients with cSCC.

## 1. Introduction

Cutaneous squamous cell carcinoma (cSCC) is the second most common form of skin cancer, with an increasing incidence worldwide [1,2]. This trend is particularly concerning due to the potential for metastatic spread in a subset of cases. While the vast majority of cSCC patients are successfully treated with surgical excision, those who develop metastatic disease may face life-threatening complications [3]. The challenge lies in accurately identifying which of these patients have cancer with a higher metastatic risk, as current management guidelines based on clinical and histopathological features often lack the necessary precision [4,5,6].

Risk stratification in cSCC commonly relies on staging systems such as the American Joint Committee on Cancer (AJCC) 7th and 8th editions, the Brigham and Women’s Hospital (BWH) system, and the NCCN guidelines [5,7,8,9]. However, these approaches have demonstrated limitations in predicting metastatic risk. Studies have revealed a troubling pattern of misclassification, with approximately 30% of cases that ultimately develop metastasis categorized as low T stage, while over 70% of cases classified as high-risk T stage do not progress to metastasis [5]. This imprecision in risk stratification has far-reaching implications, potentially leading to both under-treatment of high-risk patients and over-treatment of those with less aggressive diseases.

Considering these challenges, efforts to enhance tumor staging and risk stratification of cancers have driven interest in more sophisticated methods of tumor analysis. One method in particular, gene expression profiling, offers intriguing clinical utility for a multitude of cancers. Gene expression profiling (GEP) comprises a powerful technique that simultaneously measures the activity of thousands of genes and provides a comprehensive analysis of cellular function at the molecular level [10]. In the field of oncology, GEP has emerged as a valuable tool for tumor classification, prognosis prediction, and treatment selection across various cancer types [11]. Its ability to capture the complex molecular landscape of tumors offers the potential for more nuanced and accurate risk assessment. Proof of principle has already been demonstrated for cutaneous melanoma, for which a 31-gene GEP analysis of 901 melanomas demonstrated improved risk stratification within each AJCC v8 stage [12]. A larger cohort of over 4000 melanoma patients with linked SEER and 31-GEP results also substantiated the potential to refine prognosis based on molecular risk profile [13].

Specifically, for cSCC, a 40-gene expression profile (40-GEP) test has been developed to address the limitations of current staging systems. This innovative test analyzes the expression of 40 genes that have been implicated in cSCC progression and metastasis [14]. The selection of these genes, based on insights from molecular pathways involved in cSCC pathogenesis, includes genes related to cell cycle regulation, DNA repair, and the tumor microenvironment [14]. By capturing this diverse array of molecular markers, the 40-GEP test aims to address the shortcomings in staging and provide a more comprehensive assessment of tumor biology and metastatic potential.

The recent literature has begun to support the integration of GEP in cSCC management. A meta-analysis by Masarwy et al. (2023) suggests that incorporating GEP significantly improves risk stratification in cSCC [15]. The combination of GEP results with current staging systems has shown promise in more accurately identifying patients with an elevated risk for metastatic disease. This synergistic approach leverages both traditional clinicopathological features and molecular insights, potentially offering a more robust framework for risk assessment.

The potential impact of improved risk stratification through GEP on management decisions is substantial. Given the broad nature of current management guidelines, a more refined approach to risk assessment could lead to more personalized and effective treatment strategies. This could manifest in various ways, including adjustments in follow-up frequency, tailored imaging schedules, and the judicious consideration of adjuvant therapies for patients identified as high-risk for metastasis [16,17,18,19]. By potentially reducing unnecessary treatments for low-risk patients and ensuring adequate monitoring and intervention for high-risk individuals, GEP could contribute to both improved patient outcomes and more efficient resource allocation in healthcare systems responsible for treating these patients.

The purpose and extent of this review is to comprehensively assess the current literature on the role of gene expression profiling, with a particular focus on the 40-GEP test, in the management of cSCC. We aim to explore its prognostic value, evaluate its clinical utility, and discuss the potential implications for future management strategies. By synthesizing the available evidence, this review seeks to provide clinicians and researchers with a thorough understanding of the current state of GEP in cSCC management and to highlight areas for future research and clinical application. As the field of personalized medicine continues to evolve, the integration of molecular profiling techniques like GEP may represent a significant step forward in optimizing care for cSCC patients.

## 2. Cutaneous Squamous Cell Carcinoma

Cutaneous squamous cell carcinoma is a significant public health concern, with its incidence steadily rising worldwide [20,21]. As the second most common form of skin cancer after basal cell carcinoma, cSCC accounts for approximately 20% of all skin cancers, underscoring its importance in dermatological oncology [22,23]. This increasing incidence can be attributed to several interrelated factors that reflect both demographic shifts and changing lifestyle patterns. One of the primary drivers of the rising cSCC incidence is the aging population. The risk of developing cSCC increases substantially with age, and as global life expectancy continues to rise, more cases are being diagnosed among older individuals [21]. This trend is particularly pronounced in developed countries where the proportion of elderly citizens is growing rapidly, highlighting the need for targeted prevention and screening strategies for this demographic [24]. The impact of this, among other factors, is particularly evident in the United States, where over 1 million cases of cSCC are diagnosed annually, with the incidence increasing by 2–4% each year [2]. This steady rise represents a significant healthcare challenge, underscoring the need for effective prevention strategies, refined risk assessment tools, and personalized treatment approaches.

### 2.1. Increasing Incidence

The rising incidence of cSCC is a global phenomenon observed across different geographic regions and ethnicities. A systematic review by Lomas et al. (2012) reported significant increases in cSCC incidence in Europe, North America, and Australia over the past several decades [20]. For example, the age-standardized incidence rate in the Netherlands increased from 22.2 to 35.4 per 100,000 person-years between 1989 and 2008 [23,25]. Further, in an analysis of the Global Burden of Disease Study from 1990 to 2017, squamous cell carcinomas had the greatest increase in prevalence across all neoplasms tracked in the study [24]. Similarly, in the United States, a study using Medicare data showed a 100% increase in cSCC procedures between 1992 and 2012 [2]. This growing burden highlights the importance of accurate risk stratification and management strategies for cSCC.

Outside of ultraviolet radiation exposure, other risk factors that have been associated with the development of cSCC include immunosuppression, pre-existing lesions, and novel targeted therapies. With respect to immunosuppression, while global immunosuppression rates are not widely reported, a recent study using the US National Health Interview Survey database estimates that the prevalence of immunosuppression has risen in the United States from ~3% in 2013 to ~6% in 2021 [26,27]. Several relatively new medications used for oncologic treatment such as vismodegib and BRAF inhibitors have also been associated with the development of cSCC [28,29]. Human papillomavirus, while a more well-recognized risk factor for mucosal squamous cell carcinomas, has also been hypothesized to be associated with the development of cSCC [30].

cSCC is characterized by significant genetic heterogeneity, with multiple genes and pathways implicated in its development and progression. The tumor suppressor gene TP53 is frequently mutated in cSCC, with alterations found in up to 90% of cases, often resulting from UV-induced DNA damage [31]. The NOTCH signaling pathway, crucial for epidermal differentiation, is also commonly affected, with inactivating mutations in NOTCH1 and NOTCH2 suggesting their tumor suppressor role in the skin [32,33]. CDKN2A, which encodes the p16 tumor suppressor protein, is frequently inactivated in cSCC through mutation or promoter hypermethylation [34,35]. Other genes frequently altered in cSCC include members of the RAS family (HRAS, KRAS, NRAS), with activating mutations found in a subset of cases [36]. Mutations in PTCH1, part of the Hedgehog signaling pathway, have also been associated with cSCC development, particularly in the context of basal cell nevus syndrome [37]. The genetic heterogeneity of cSCC underscores the potential value of comprehensive molecular profiling techniques like gene expression profiling in improving risk stratification and guiding personalized management strategies.

### 2.2. High-Risk cSCC

The concept of high-risk cSCC has evolved as our understanding of prognostic factors has improved. Current definitions of high-risk cSCC incorporate a range of clinical, histopathological, and patient-specific factors. Tumor characteristics associated with high risk by most guidelines include size greater than 2 cm in diameter, invasion beyond subcutaneous fat or depth greater than 6 mm, location in high-risk areas, poorly defined borders, and rapid growth or associated symptomatology such as pain, numbness, or paresthesia that may indicate perineural invasion [3,38,39,40,41]. Histopathological features indicative of high risk include poor or undifferentiated histology, acantholytic, adenosquamous, or desmoplastic subtypes, perineural or lymphovascular invasion, and the presence of in-transit metastases. Some patient factors that contribute to high-risk classification include immunosuppression, a history of radiation therapy to the affected area, or recurrent lesions. The NCCN also separates high-risk and very-high-risk cSCC, as indicated in Table 1 [39,40].

The statistics associated with high-risk cSCC emphasize the importance of accurate identification and appropriate management of these cases. While the overall metastasis rate for cSCC is approximately 2–5%, this rate can increase to 10–20% for tumors classified as high risk [42,43,44]. Similarly, while the overall disease-specific mortality for cSCC is relatively low at 1–2%, patients with metastatic disease face a much poorer prognosis, with 5-year survival rates dropping to 25–35%. [43,45]. Local recurrence rates for high-risk cSCC are also concerning, ranging from 10 to 50% depending on the specific risk factors present and the treatment/management approach [45,46,47].

Guideline-based treatments for high-risk cSCC are summarized in Table 2 [39,40,41,48]. Adjuvant radiation therapy (ART) is broadly considered for such patients. Recently, a multidisciplinary panel consisting of radiation oncologists and dermatologists/Mohs micrographic surgeons with expertise in cSCC management met to discuss the potential utility of 40-GEP testing to guide clinical decision-making with respect to adjuvant radiation therapy [49]. The panel specifically recommended consideration of ART for patients with specific aggressive classes based on 40-GEP testing.

## 3. Gene Expression Profiling

Gene expression profiling (GEP) has emerged as a powerful tool in oncology, providing valuable insights into tumor biology, prognosis, and treatment response across various cancer types. In breast cancer, the 21-gene Oncotype DX and 70-gene MammaPrint assays are widely used to guide adjuvant therapy decisions in early-stage disease [11,50]. For prostate cancer, tests like Decipher and Prolaris employ GEP to assess tumor aggressiveness and inform treatment decisions [51,52]. In melanoma, the 31-gene expression profile test (DecisionDx-Melanoma) is used to predict metastatic risk. At the same time, in colon cancer, the 12-gene Oncotype DX Colon Recurrence Score assesses recurrence risk in stage II patients [53,54]. These examples demonstrate the broad applicability of GEP in oncology and its potential to improve personalized cancer management.

The 40-gene expression profile (40-GEP) test for cutaneous squamous cell carcinoma (cSCC) was developed to address the need for more accurate risk stratification in this cancer type. Through a comprehensive literature review and analysis of cSCC genomics data, researchers identified genes involved in key biological processes relevant to cSCC progression, including cell cycle regulation, DNA repair, epithelial-to-mesenchymal transition, and immune response [14]. Machine learning techniques were employed to develop an algorithm that could classify tumors into risk categories based on the expression patterns of the selected genes, and the test underwent further validation in independent cohorts to assess its prognostic accuracy and clinical utility [14,17,18,55,56,57].

The 40-GEP test classifies cSCC tumors into three risk classes: class 1 (low risk), class 2A (higher risk), and class 2B (highest risk) [14]. This classification is based on the collective expression pattern of 34 discriminant and 6 control genes, providing a more comprehensive assessment of tumor biology than traditional clinicopathological factors alone [14]. While the complete list of genes included in the 40-GEP test was derived through both literature review as well as transcriptomic analysis, some of the key genes included in the 40-GEP test were found to be associated with metastasis in multiple primary research studies including MMP10 (Matrix Metalloproteinase 10), MRC1 (Mannose Receptor C-Type 1), and PI3 (Peptidase inhibitor 3) [14,58,59,60,61].

These genes, along with others in the 40-GEP, provide a comprehensive picture of the tumor’s biological behavior, including its potential for invasion, metastasis, and immune evasion. By analyzing the expression patterns of these genes collectively, the 40-GEP test aims to provide a more accurate assessment of metastatic risk than traditional staging systems alone. The development and validation of the 40-GEP test represents a significant step towards personalized medicine in cSCC management, potentially allowing for more tailored treatment and follow-up strategies based on individual tumor biology.

## 4. Usage of GEP and Prognostic Performance

The prognostic value of the 40-gene expression profile (40-GEP) test in cutaneous squamous cell carcinoma (cSCC) has been extensively studied. Several studies have explored the integration of the 40-GEP test into guideline-driven care for high-risk cSCC. One study by Arron et al. investigated the risk of metastatic disease progression and compared ART-treated and untreated patients. They found that for patients with GEP class 2B disease, their risk of and time to local recurrence or metastasis were increased compared to other patients, and further, for the subset of class 2B patients who underwent ART, their disease progression became similar to those of patients with class 2A disease [62]. Wysong et al. created multivariable models integrating 40-GEP results alongside individual clinicopathologic risk factors with the BWH, AJCC 8th edition risk classification system, and NCCN; in multivariate analysis, they demonstrated that GEP classes 2A and 2B were independent risk factors with their inclusion leading to improved positive and negative predictive values for metastasis within guideline-based risk categories [63]. In another study investigating head and neck cSCC specifically, analysis of 278 tumor samples found a 3-year metastasis-free survival rate of 92.1% for class 1 patients, with 76.1% and 44.4% for class 2A and class 2B patients, respectively, with multivariate analysis demonstrating the independent prognostic value of GEP classification [56]. The results of four articles wherein the independent prognostic capability of the 40-GEP test in multivariate analyses incorporating clinicopathologic characteristics is shown below in Table 3.

A systematic review and meta-analysis by Masarwy et al. (2023) provide a comprehensive assessment of the test’s performance, including 1019 patients from three cohort studies. The analysis revealed 3-year metastasis-free survival rates of 92.4%, 78.9%, and 45.4% for class 1, class 2A, and class 2B, respectively [15]. Notably, the positive predictive value for metastasis was significantly higher for class 2B compared to the American Joint Committee on Cancer 8th edition (AJCC8) and Brigham and Women’s Hospital (BWH) staging systems [5,7,15].

In summary, the available evidence suggests that the 40-GEP test provides additional prognostic value beyond traditional staging systems and other prognostic tools. Its integration into clinical practice, in combination with other risk assessment methods, could lead to more personalized and effective management strategies for cSCC patients. The ongoing research in this area continues to refine our understanding of how best to incorporate the 40-GEP test into clinical decision-making processes, potentially improving outcomes for patients with high-risk cSCC.

## 5. Impact on Clinical Decision-Making

Several studies have investigated how the results of the 40-gene expression profile (40-GEP) test influence clinical decision-making in cutaneous squamous cell carcinoma (cSCC) management. In a study conducted by Hooper et al. (2022), clinicians who ordered ten or more 40-GEP tests during the first year the test was clinically available were surveyed. The study found that physicians were appropriately ordering the test for high-risk patients. When presented with example cases of high-risk cSCC and subsequently a GEP class stratification, changes in management plans were aligned with the level of risk. De-escalation of management was observed after presentation with a class 1 result, while intensification of management occurred with a class 2B result [57]. Other studies have administered surveys to physicians managing cSCC patients with clinical vignettes and recording proposed management before and after receiving GEP class results, finding that low-risk GEP class was associated with a risk-aligned decrease in adjuvant radiation or chemotherapy, imaging, and sentinel lymph node biopsy with the inverse being true for the highest risk class [16,64].

The main areas of cSCC management influenced by GEP testing results include adjuvant therapy decisions, follow-up protocols, sentinel lymph node biopsy considerations, and referral patterns. Across studies, surveyed clinicians demonstrated consistent risk-aligned decision-making within the broad treatment guidelines for increases and decreases in recommendations for ART and more/less frequent follow-up visits, especially for patients with class 1 and class 2B results [16,64,65,66]. Recently, a multidisciplinary panel convened to discuss the integration of clinicopathologic staging systems with 40-GEP testing results with regard to metastatic risk stratification and ART, ultimately providing a clinical workflow wherein patients who have at least one or more high-risk features and a GEP result of high (2A) or highest (2B) risk for metastasis are referred for ART and have a discussion of its risks and benefits following primary surgical management per NCCN guidelines [49]. Within each AJCC stage, the class 2B risk profile was associated with a 5- to 10-fold higher risk of metastasis within 3 years and was perceived as a strong indication for ART regardless of the presence or absence of other high-risk features [49]. The class 2A risk profile was associated with two- to three-fold higher 3-year metastatic risk, and ART is also considered/recommended [44]. This integrated approach shows promise in allowing physicians to counsel their patients in a more risk-aligned manner, potentially moving towards more personalized medicine. By combining traditional staging methods with molecular profiling, clinicians can more accurately assess an individual patient’s risk and tailor treatment recommendations accordingly. While this test is still in its early phases, this paradigm could enable more nuanced care decisions, potentially allowing for escalation of care for those patients at the highest risk, even if classified as AJCC-T1, and a more measured approach for patients with traditionally high-risk features (such as AJCC-T3) but demonstrating a low-risk GEP class.

Integrating the 40-GEP test into clinical practice has demonstrably influenced decision-making in cSCC management. The test enables more personalized treatment approaches by providing additional risk stratification beyond traditional staging systems. This is particularly evident in decisions regarding adjuvant therapy and follow-up intensity. The consistency of risk-aligned decision-making across multiple studies suggests that the 40-GEP test can become a valuable tool in the clinical arsenal for the management of high-risk cSCC. However, as with any novel prognostic tool, ongoing research, and more real-world evidence will be crucial in further validating its impact on patient outcomes and refining its role in broader clinical practice.

## 6. Limitations

There are some potential barriers and limitations to the widespread use of the 40-GEP test. When any new prognostic test is developed, the chief concern is whether the use of the test is justified by its benefit in targeting treatments towards the patients most at risk of negative outcomes. While real-world evidence of this is currently unavailable in the literature, recently, Somani et al. utilized a commercial claims database to estimate the cost of ART for Medicare patients with cSCC and performed analyses to determine the potential cost savings through avoidance of ART for patients determined to be at low or intermediate risk by GEP testing at various distributions, reporting potential savings of USD 144–972 million [67]. Despite these potential population-level cost savings, it should be kept in mind that undergoing 40-GEP testing is costly and may not be covered by some insurers, with estimates at our institution ranging from USD 3000 to 7000, and insurance reimbursement of USD ~600 [68]. This clearly represents a hurdle to the accessibility of the test for the broader population, particularly in light of the fact that there is inconsistent Medicare coverage of the 40-GEP assay.

It should also be noted that despite the body of evidence that the GEP test demonstrates improved stratification of metastatic risk, as previously discussed, the likelihood of cSCC patients who are treated appropriately developing regional or distant metastases is quite low, with estimates in the range of ~1.2–5% [69,70]. While epidemiologic data on cSCC are somewhat limited due to their absence or limited data collected in various national cancer registries, a recent prospective analysis of 1400 cSCC patients found that 2.4% of deaths in their cohort were due to cSCC, with those deaths attributed quite evenly between distant metastasis, locoregional metastases, and local infiltration (30–36%); this ultimately limits the utility of the 40-GEP, which is only validated in the prediction of risk of metastasis [45,71].

The 40-GEP test employs machine learning algorithms to generate a continuous probability of metastasis risk, which is subsequently categorized into discrete risk classes (classes 1, 2A, and 2B) [14]. When considering melanoma, the Melanoma Prevention Working Group reached a consensus in 2020 that the results of GEP testing should be viewed as continuous, aligning with the algorithms and biological realities underlying such tests [72]. The discretization of a continuous risk model into categories, albeit pragmatic for clinical decision-making and patient education, could mask nuances or exaggerate differences in individual patient risk profiles. Clinicians should be aware that patients near the category boundaries may have similar risk profiles despite being classified differently. Future research should focus on optimizing these risk thresholds and potentially developing more nuanced risk stratification models. Public development of a more nuanced multivariate risk stratification model and/or nomogram incorporating clinicopathological variables along with 40-GEP may ultimately prove to be the most accurate approach for risk stratification in cSCC patients [17,55,56,63].

## 7. Implications for Current and Future Management

The integration of gene expression profiling (GEP) into managing cutaneous squamous cell carcinoma (cSCC) has significant implications for current and future clinical practice. These implications stem from the improved risk stratification that GEP provides and its potential to guide more personalized treatment approaches.

GEP testing allows for more nuanced risk stratification than traditional staging systems alone, which could lead to tailored follow-up protocols. Highest risk patients (class 2B) may benefit from more intensive surveillance and ART [17,18,49,62,73]. Conversely, patients classified as lowest risk (class 1) may be able to safely forego ART and undergo less intensive surveillance, potentially reducing healthcare costs and patient burden [49]. GEP results could also help identify patients who are most likely to benefit from adjuvant radiation therapy or systemic treatments and could aid in identifying high-risk patients suitable for clinical trials of novel therapies or treatment intensification strategies.

The future of cSCC management likely involves the integration of multiple prognostic tools. This may include developing and validating risk assessment models that incorporate GEP results alongside traditional clinicopathologic factors and other molecular markers. Artificial intelligence algorithms could potentially analyze GEP data alongside other clinical and pathological features to provide more comprehensive risk assessments, as has been carried out using the i31-GEP algorithm in cutaneous melanoma [74].

Understanding the gene expression patterns in high-risk cSCC could lead to new therapeutic targets. Identifying overexpressed genes or activated pathways in high-risk tumors could guide the development of targeted therapies. GEP data might also help identify patients who are more likely to respond to immunotherapies, potentially by identifying immune-related gene signatures. As has already been discussed, there is a movement in the dermatology and radiation oncology fields on utilizing this test in order to focus on adjuvant radiation therapy for those patients at the highest risk of metastasis [49].

Incorporating GEP testing into routine clinical practice has economic implications that need to be considered. Future research should focus on determining the cost-effectiveness of routine GEP testing in cSCC management. As evidence for the clinical utility of GEP testing accumulates, there may be changes in insurance coverage and reimbursement policies.

## 8. Conclusions

While the current evidence is promising, several areas require further investigation. Studies with longer follow-up periods are needed to assess the impact of GEP-guided management on long-term survival and quality of life. Prospective randomized trials comparing GEP-guided management to current guideline-driven care are necessary to definitively establish the clinical utility of GEP testing. Further research into the biological significance of the gene expression patterns identified by GEP could lead to new insights into cSCC pathogenesis and progression.

In conclusion, while GEP testing, particularly the 40-GEP test, shows significant ability in improving risk stratification and guiding personalized management in cSCC, several important limitations and concerns exist. Ongoing research, including large-scale prospective studies and real-world implementation analyses, will be crucial in addressing these issues and optimizing the use of GEP in clinical practice. As the field evolves, continuous reassessment of the role of GEP testing in cSCC management will be necessary to ensure its appropriate and effective utilization.

## Figures and Tables

**Table 1:** A comparison of the European Association of Dermato-Oncology (EADO) and National Comprehensive Cancer Network (NCCN) guidelines for high-risk cutaneous squamous cell carcinoma. + indicates that the presence of that variable is considered high-risk; - indicates that the variable is not considered when determining high-risk status.

Variable	EADO [38]	NCCN [39]
Intrinsic		
Size	>2 cm	High risk: >2 and <4 cm Very high risk: >4 cm
Location	Temple, ear, lip	Head, neck, hands, feet, pretibial, anogenital area
Depth of invasion	>6 mm or beyond fatty tissue	>6 mm or beyond fat tissue
Perineural invasion	Microscopic, symptomatic, radiological	High risk: + Very high risk: Tumor cells within the nerve sheath of a nerve lying deeper than the dermis or measuring ≥0.1 mm
Degree of differentiation	Poor differentiation	Poor differentiation
Desmoplasia	+	High-risk subtypes: Acantholytic, adenosquamous, metaplastic Very high risk: +
Growth rate	-	+ rapidly growing tumor
Bone erosion	+	-
Borders	-	+ poorly defined
Lymphatic or vascular involvement	-	High risk: - Very high risk: +
Extrinsic factors		
Primary vs. recurrent	-	Recurrent
Prior radiotherapy	-	+
Immunosuppression	+	+

**Table 2:** Overview of guideline-driven care for high-risk cSCC. Abbreviations: American Academy of Dermatology (AAD), American Society for Radiation Oncology (ASTRO), National Comprehensive Cancer Network (NCCN), American Joint Committee on Cancer (AJCC), adjuvant radiation therapy (ART), peripheral nerve invasion (PNI).

Treatment	AAD [40]	ASTRO Task Force [41]	NCCN [39,48]
ART to primary site	Recommends: - For concerning PNI and/or high risk for regional or distant metastasis	Strongly Recommends: - For clinically or radiologically evident gross PNI - When further procedures cannot correct or close positive margins - When recurrence follows a margin-negative resection - For AJCC8 T3 and T4 tumors - For chronically immunosuppressed patients with desmoplastic or infiltrative tumors	Recommends: - For extensive PNI or with large (nerve caliber ≥ 0.1 mm) nerve involvement
Chemotherapy	N/A	N/A	Recommends: - Against the use of systemic therapy for local diseases amenable to surgery - Potential use with ART when further surgery is not an option - Consideration for regional recurrence if the patient is ineligible for immunotherapy and clinical trials
Immunotherapy and targeted therapy	N/A	N/A	Recommends: - Against the use of systemic therapy for local diseases amenable to surgery - Potential use of immunotherapy with RT in the clinical trial context for residual disease in locally advanced cSCC when further surgery is not an option or in complicated cases when curative surgery and RT are not feasible - Immunotherapy with a checkpoint inhibitor is preferred in regional recurrence when curative surgery and RT are not feasible

**Table 3:** Summary table of select studies in which GEP class was assessed in multivariate models incorporating existing staging systems and/or clinicopathologic variables. Abbreviations: cutaneous squamous cell carcinoma (cSCC); metastasis-free survival (MFS); National Comprehensive Cancer Network (NCCN); American Joint Committee on Cancer (AJCC); Brigham and Women’s Hospital tumor classification system (BWH); gene expression profiling (GEP).

Author/Year	Population	Univariate Analysis	Multivariate Analysis
Wysong et al. 2021 [63]	Localized high-risk cSCC	Class 1: 91.6% 3-year MFS Class 2A: 80.6% 3-year MFS Class 2B: 44.0% 3-year MFS	Independent predictor of risk in bivariate models with AJCC or BWH T stage. Multivariable analysis with clinical features prior to surgery demonstrated independent significance of class 2A and class 2B. Multivariable analysis with histopathologic variables demonstrated the independent significance of class 2B.
Ibrahim et al. 2021 [55]	cSCC with ≥ 1 high-risk feature per NCCN guidelines or AJCC/BWH T > 1	Class 1: 93.9% 3-year MFS Class 2A: 80.5% 3-year MFS Class 2B: 47.8% 3-year MFS	When incorporated into a multivariate model with clinicopathologic variables, GEP class 2A and class 2B statuses were both independently associated with metastatic risk, with class 2B conferring the highest hazard ratio of included variables.
Arron et al. 2022 [56]	Head and neck cSCC	Class 1: 92.1% 3-year MFS Class 2A: 76.1% 3-year MFS Class 2B: 44.4% 3-year MFS	Multivariate analysis with AJCC8 or BWH T stage, as well as clinicopathologic risk factors, demonstrated independent risk prediction of the GEP class.
Moody et al. 2024 [17]	High-risk cSCC	N/A	Hierarchical cluster analysis to determine clinicopathologic profiles of patients who received ART. Matched-pair analysis with random resampling within the GEP class showed significant improvement in 5-year MFS for class 2B patients receiving ART. There was no accordant improvement in MFS for class 1 patients.
